# Supplementary figures and images for: A genetic toolkit for studying transposon control in the Drosophila melanogaster ovary
Source: Genetics. 2021 Oct 30;220(1):iyab179. doi: 10.1093/genetics/iyab179 (PMC8733420; doi:10.1093/genetics/iyab179)

# FIGURE S1

A

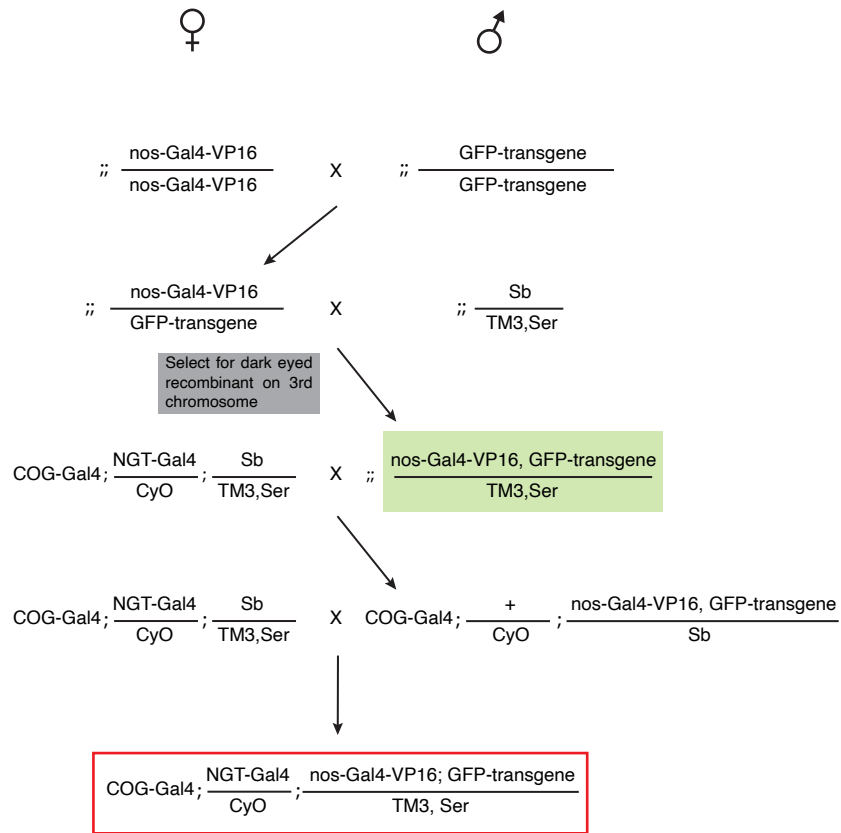

B

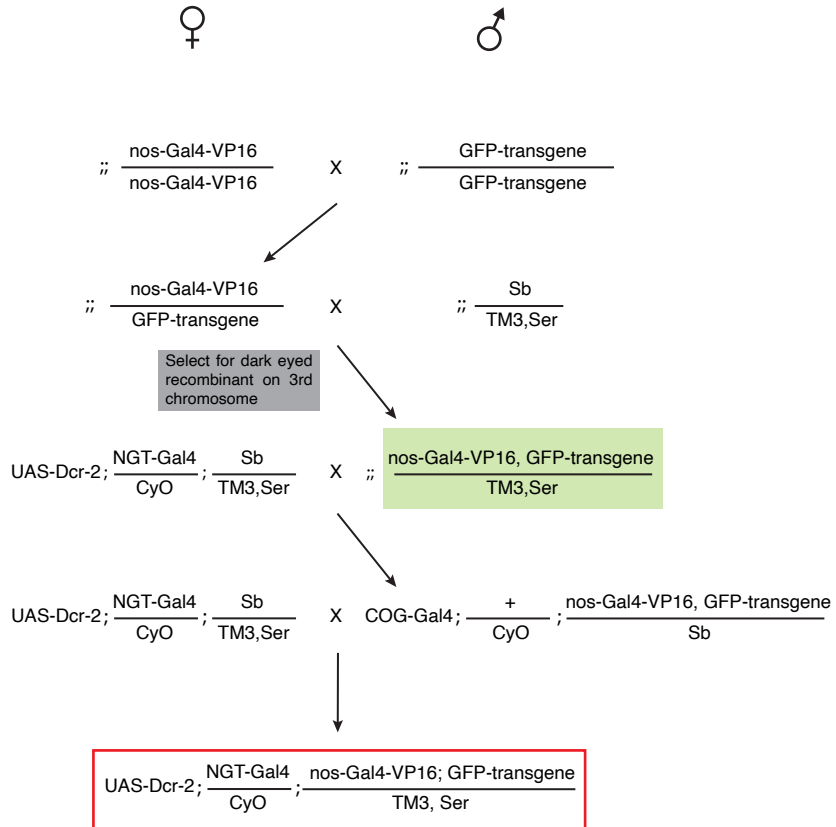

Supplement: iyab179_Supplementary_Figure_1 [file iyab179_supplementary_figure_1.pdf]
