# Supplementary material for: A genetic toolkit for studying transposon control in the Drosophila melanogaster ovary
Source: Genetics. 2021 Oct 30;220(1):iyab179. doi: 10.1093/genetics/iyab179 (PMC8733420; doi:10.1093/genetics/iyab179)
Supplement: iyab179_Supplementary_Figure_1_Caption [file iyab179_supplementary_figure_1_caption.docx]

**Figure S1: Crossing scheme to generate marker lines with Gal4 Drivers.**

(A) Scheme for construction of MTD-Gal4 lines with compatible with short hairpin RNA (shRNA) UAS-lines.

(B) Scheme for construction of nanos-Gal4 lines with a UAS-Dcr-2 transgene compatible with long hairpin RNA UAS-lines.
